# Patient-Facing Mobile Apps to Support Physiotherapy Care: Protocol for a Systematic Review of Apps Within App Stores

**DOI:** 10.2196/29047

**Published:** 2021-12-09

**Authors:** Mark Merolli, Jill J Francis, Patrick Vallance, Kim L Bennell, Peter Malliaras, Rana S Hinman

**Affiliations:** 1 Centre for Health Exercise and Sports Medicine, Department of Physiotherapy School of Health Sciences The University of Melbourne Melbourne Australia; 2 Centre for Digital Transformation of Health The University of Melbourne Melbourne Australia; 3 School of Health Sciences The University of Melbourne Melbourne Australia; 4 Department of Physiotherapy, School of Primary and Allied Health Care Faculty of Medicine, Nursing and Health Science Monash University Melbourne Australia

**Keywords:** physiotherapy, physical therapy, digital health intervention, mobile app, eHealth, behavior change technique, behavior change, exercise, digital health, mHealth

## Abstract

**Background:**

Care delivered by physiotherapists aims to facilitate engagement in positive health behaviors by patients (eg, adherence to exercise). However, research suggests that behavioral interventions are frequently omitted from care. Hence, better understanding of strategies that can be used by physiotherapists to support patients to engage in positive behaviors is important and likely to optimize outcomes. Digital health interventions delivered via mobile apps are garnering attention for their ability to support behavior change. They have potential to incorporate numerous behavior change techniques (BCTs) to support goals of physiotherapy care, including but not limited to self-monitoring, goal setting, and prompts/alerts. Despite their potential to support physiotherapy care, much is still unknown about what apps are available to consumers, the BCTs they use, their quality, and their potential to change behaviors.

**Objective:**

The primary aim of this study is to systematically review the mobile apps available in app stores that are intended for use by patients to support physiotherapy care, including the BCTs within these apps. The secondary aims are to evaluate the quality and behavior change potential of these apps.

**Methods:**

A systematic review of mobile apps in app stores will be undertaken. This will be guided by recommendations for systematic reviews in line with the PRISMA (Preferred Reporting Items for Systematic Reviews and Meta-Analyses) statement but adapted to suit our app store search, consistent with similar systematic reviews of apps published in the *Journal of Medical Internet Research*. Apple Store and Google Play will be searched with a two-step search strategy, using terms relevant to physiotherapy, physiotherapists, and common physiotherapy care. Key eligibility criteria will include apps that are intended for use by patients and are self-contained or stand-alone without the need of additional wearable devices or other add-ons. Included apps will be coded for BCTs and rated for quality using the Mobile Application Rating Scale (MARS) and for potential to change behavior using the App Behavior Change Scale (ABACUS).

**Results:**

App store search and screening are expected to be completed in 2021. Data extraction and quality appraisal are expected to commence by November 2021. The study results are expected to be published in a subsequent paper in 2022.

**Conclusions:**

Knowledge gained from this review will support clinical practice and inform research by providing a greater understanding of the quality of currently available mobile apps and their potential to support patient behavior change goals of physiotherapy care.

**International Registered Report Identifier (IRRID):**

PRR1-10.2196/29047

## Introduction

Care delivered by physiotherapists involves a complex mix of biopsychosocial components that aim to facilitate engagement in positive health behaviors by patients, such as adherence to prescribed exercise programs or greater participation in physical activity [1-5]. Facilitating positive behavior change is particularly important as care moves to the home or community, where patients typically spend increasing time without physiotherapist supervision [2]. To promote this, various strategies that support patient engagement in desired behaviors may be used by the physiotherapist, such as education, provision of a home exercise program as a component of self-management, demonstration of these exercises, and instructional information about how to perform the behavior [1,3]. Despite this, research suggests that positive patient behaviors are suboptimal and that behavioral interventions are frequently omitted from physiotherapy care [3]. Thus, strategies that support patients to achieve and maintain positive behavior change over the long-term, particularly where supervision from the physiotherapist has ceased, are important and likely to optimize treatment benefits [1,3].

Digital health interventions are gaining increasing attention in various physiotherapy contexts [6-13]. They are defined as “...interventions delivered via digital technologies...to provide effective, cost-effective, safe, and scalable interventions to improve health and healthcare” [14]. These technologies include but are not limited to the internet, smartphones, wearables, and other connected devices. Mobile apps are a form of digital health interventions that are receiving increasing attention for supporting care and driving behavior change [15], including patient behaviors such as self-management activities (eg, exercise participation) [7,16-20]. A key strength of mobile apps is their potential to support patients’ ongoing behavioral performance during and between consultations, or when formal treatment has ceased. They are widely available, inexpensive, and scalable [21]. Apps have the capacity to deliver numerous behavior change techniques (BCTs) conducive to physiotherapy care. BCTs are defined as “an observable, replicable, and irreducible component of an intervention designed to alter or redirect causal processes that regulate behavior” [22]. These techniques refer to the “active ingredients” of the intervention, which facilitate the intended behaviors one wishes to change [2,23]. BCTs can be further unpacked using a clustering taxonomy, the Behavior Change Technique Taxonomy version 1 (BCTTv1), which was developed to create a more robust system for reporting about behavior change interventions [22]. In this digital context, these interventions may include but are not limited to self-monitoring, goal setting, prompts/alerts, social support, feedback, action planning, rewards, scheduling, instructional information, and social support [7,10,15,18]. The research landscape surrounding mobile apps and health behavior change is still maturing, and to date, there is no agreement on how to facilitate behavior change. Research still seeks to understand which components of apps may best facilitate behavior change in the user. Further, there is a dearth of evidence regarding the features of apps that are most conducive to delivering evidence-based BCTs, as well as how to evaluate and categorize these features [15]. It was for this reason that the authors McKay, Slykerman, and Dunn [15] developed the App Behavior Change Scale (ABACUS) with the aim to support the assessment of the behavior change potential of mobile apps through quantification of BCTs.

Specifically for physiotherapy, little is currently known about the range of apps available on the market to support physiotherapy care, the quality of these apps, and their potential to change behavior. Hence, the objectives of this review are to catalogue the apps (intended for use by patients) that are available in app stores, the BCTs they contain, their quality, and their potential to change behavior. Our specific research question is “What is the quality of mobile apps designed for patients to support physiotherapy care, and what BCTs do they contain?”

## Methods

### Study Design

The proposed study is a systematic review of mobile apps available in app stores that are intended for use by patients to support physiotherapy care. This review will be guided by principles for systematic reviews in line with the PRISMA (Preferred Reporting Items for Systematic Review and Meta-Analyses) statement [24] but will be adapted to suit a search of app stores, which differs from searches of published or grey literature for most systematic reviews. This approach is well-grounded in published literature in this journal, in which systematic reviews were conducted examining mobile apps within app stores [7,25]. The search will be developed in line with the 2015 PRISMA-P (Preferred Reporting Items for Systematic Review and Meta-Analysis Protocols) checklist for developing a systematic review protocol [26].

### Study Objectives

Our study objectives are as follows.

#### Primary

The primary objective of the study is to describe mobile apps intended for use by patients to support physiotherapy care, including a description of any BCTs contained within them (coded against the BCTTv1 taxonomy) [22].

#### Secondary

The secondary objectives of the study are (1) to evaluate app quality, using the Mobile Application Rating Scale (MARS) [27], and (2) to evaluate apps for their potential to change behavior, using the App Behavior Change Scale (ABACUS) [15].

### Ethics

This study design does not require ethical approval.

### Eligibility

The inclusion and exclusion criteria are presented in Textbox 1.

**Mobile app inclusion and exclusion criteria.**

**Inclusion criteria**

- Apps are intended for use by patients to support physiotherapy care they are receiving or have received. Note that it must be clear in either the description/title/screenshots on the app store or within the app itself that the intended use of the app is to support physiotherapy or physical therapy care (eg, alongside standard physiotherapy care, prescribed by the physiotherapist, or monitored by their physiotherapist).
- Apps are designed for self-contained/stand-alone use without the need of additional wearable devices or other add-ons (eg, wearable sensors).
- Apps are available on either the Apple App Store or Google Play platform.
- Apps are available in the English language.
- Apps will be considered irrespective of whether they are free or paid; where both exist (eg, a “lite” and full paid version), both versions will be evaluated.
- Apps will be considered irrespective of time passed since the app launch or last update, providing they are compatible with current mobile devices at the time of searching.

**Exclusion criteria**

- Apps are designed for exclusive use by health care professionals (and not by patients).
- White-labelled apps are designed for exclusive use by a specific clinic or health care service and are not available for use by the general public (eg, those requiring a unique login for that organization).
- Apps cost more than Aus $10 (US $7.33) (this is in line with other study protocols evaluating mobile health apps, as research indicates that consumers are unlikely to purchase health apps that cost more than this) [26,28].

### Sources

Because Apple (iOS) and Android devices combined accounted for 99.4% of mobile operating systems worldwide as recently as November 2020 [29], we will search their respective app platforms, the Apple App Store (Apple) and Google Play (Android). Any unique apps eligible for screening and inclusion identified through this additional search will be added.

### Search Strategy

A two-step strategy will be used to search app store platforms, consistent with the most comprehensive search strategy and study design used across other systematic reviews of health-based apps in app stores published in the *Journal of Medical Internet Research* [7]. A review of health-based apps using this two-step strategy identified 6579 apps [25], while reviews using a single-step search have typically resulted in <1000 identified apps [7,28,30]. Thus, the two-step search strategy is most appropriate and is expected to result in a greater number of identified apps, owing to differing platform search algorithms. The Apple App Store search algorithm is optimized by using a single keyword, while the Google Play store search algorithm is optimized by using string keywords [25]. For the full list of key terms searched, and both steps of the search strategy, see Textbox 2. The search will be rerun at the time of final manuscript preparation to ensure up-to-date coverage.

**Search strategy.**

**Step one:**

“physiotherapy”, “physio”, “physical therapy”, “physiotherapist”, “physical therapist”

**Step two:**

“physiotherapy”, “physio”, “physical therapy”, “physiotherapist”, “physical therapist”

**and**

“assessment”, “diagnosis”, “digital”, “eHealth”, “evaluation”, “examination”, “exercise”, “health promotion”, “intervention”, “physical activity”, “plan”, “care”, “prevention”, “rehabilitation”, “screening”, “pain”, “self-management”, “treatment”, “support”, “adherence”

In the first step, app store platforms will be searched using a predefined list of relevant key terms. This includes terms used commonly to describe physiotherapy or physiotherapists [31]. This step was based on previous reviews, which used keywords recommended by reputable sources [30]. The predefined terms used in this review have been obtained from the glossary provided by World Physiotherapy (formerly the World Confederation of Physical Therapy), the peak body for physiotherapists globally (representing >650,000 physiotherapists worldwide with 122 member organizations). This glossary was developed to support policies, guidelines, and other resources, and to aid in consistency of terminology internationally.

For the second step, app store platforms will be searched again using string keywords. This search will be performed by combining terms from step one and terms used to describe common physiotherapy care, such as “physio” and “exercise”. The terms used in step two to describe physiotherapy care are also obtained from the glossary provided by World Physiotherapy. Terms synonymous with physiotherapy care were extracted based on title and descriptions provided. The authors agreed to include additional terms (“self-management”, “pain”, “treatment”, “support”, and “adherence”), given their obvious association with physiotherapy care.

To ensure feasibility, each key term search will be limited to the first 100 apps identified, as platforms continuously refresh the end of each search list to retrieve additional apps of less relevance [25].

Additionally, websites of professional physiotherapy associations will be searched for the presence of specific pages or sections dedicated to any mobile apps that may be recommended for use by patients to support physiotherapy care. Multimedia Appendix 1 details the list of associations that will be searched, including their websites. These associations include the top 10 member organizations of World Physiotherapy based on number of members, with websites that are in English and do not require a paid membership or login to access.

### App Records

#### Selection Process

**Figure 1:**
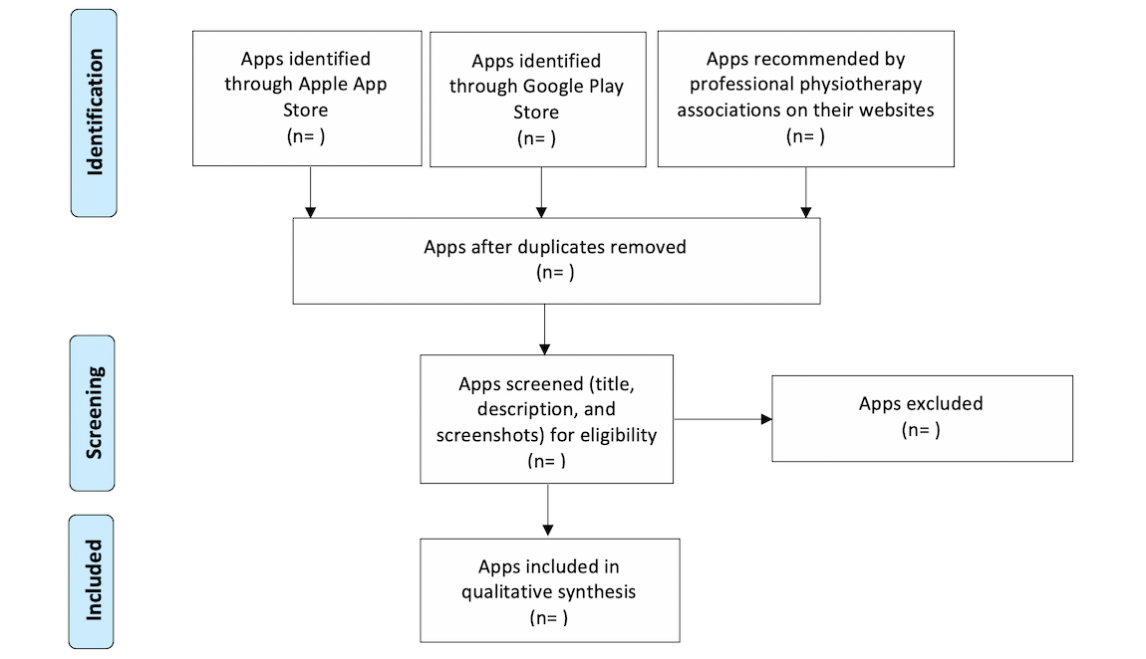
PRISMA (Preferred Reporting Items for Systematic Reviews and Meta-Analyses) flow diagram.

The review flow is shown in Figure 1. The source search, as described above, will be performed by two reviewers (MM and PV). Duplicates will be removed, and the same two reviewers will perform the screening independently across 4 devices (2 Apple; 2 Android). This search will be based on the app title, its description, and screenshots included in the app store description [32]. Any disagreements will be resolved by discussion, and where consensus is not achieved, a third reviewer (PM) will decide. The resultant apps will be downloaded to the 4 mobile devices for use by the reviewers (MM and PV) [25].

#### Extraction of Data Items

Two reviewers (MM and PV) will extract descriptive data from within the app store or within the app itself, based on information provided about the app, or within it, from its developers (ie, from the description and title of the app, “about this app” section, or any accompanying marketing or tutorial screenshots and videos). Where additional information is required (eg, whether a health care professional was involved in development), further information will be sought from the app’s source website if this is supplied in the app store description or within the app. Data will be added to an electronic spreadsheet (Excel, Microsoft Corporation) (Multimedia Appendix 2). The descriptive data to be extracted are detailed in Textbox 3.

**Descriptive characteristics that will be extracted from the included apps.**

- App name
- App developer
- Size of app (MB)
- Focus of app (specific condition/bodily region or more general, eg, low back pain; specialty, eg, neurological physiotherapy)
- Targeted behaviors if described, such as exercise (stretching, strengthening, etc), monitoring of pain (data entry), physical activity (walking, cycling, etc)
- Country of origin
- Developer qualifications (ie, health care professional or other)
- Consultation/involvement of health care professional in development
- App date of creation
- App date of update (most recent) and current version (eg, version 1, version 2)
- Cost: payment method (one-off or subscription, in-app purchases), amount (Aus $)
- Platform (Apple App Store or Google Play)
- Consumer reviews (number, ratings from 0 to 5) and times downloaded (where available)
- Any mention in the title or description of app use in published peer-reviewed literature
- Data privacy policy provided in or via the app store

Additionally, both reviewers will interact with all functions of the included apps for a minimum of 10 minutes to become familiar with the apps. The purpose of engaging with the apps and their functionalities will be to code and score the following aspects.

### BCT Identification

Reviewers will identify and code included BCTs within the apps. Coding will be conducted by two reviewers (MM and PV) who have completed web-based training to receive certification for recognizing and coding BCTs [33]. This web-based training is based on a tutorial session model that improved coder agreement with expert consensus, confidence to assess BCTs, and coding competence [34]. A third experienced behavior change expert reviewer (JF), who was one of the original creators of the BCTTv1 and hence an expert in BCTs, will be involved to resolve any disagreements between the reviewers (MM and PV) via discussion.

**Figure 2:**
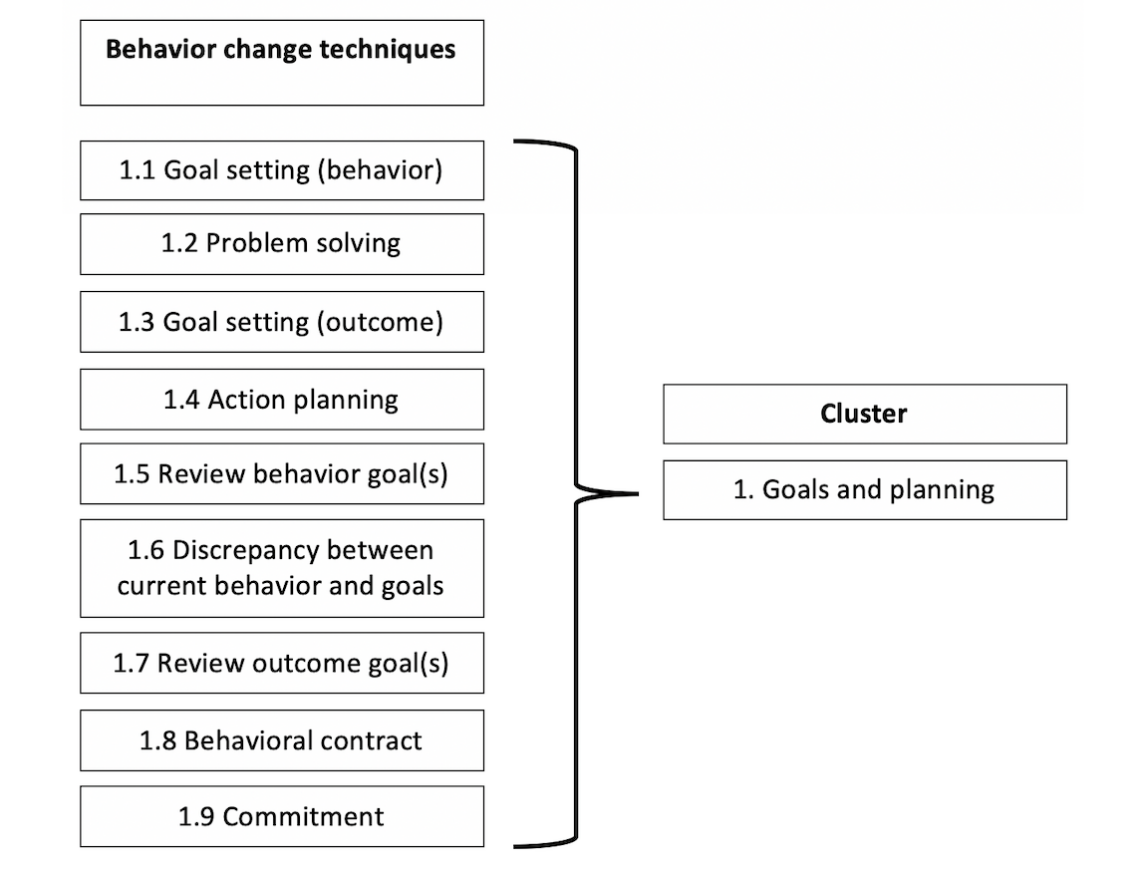
Example of behavior change techniques organized into their corresponding cluster [22].

Based on identified target behaviors within included apps, the reviewers will independently identify BCTs and categorize them into corresponding clusters in line with the BCTTv1 (Multimedia Appendix 3) [22]. The BCTTv1 is a framework of 93 clustered BCTs that was developed to ensure methodological rigor for the reporting on behavior change interventions. Using the BCTTv1, BCTs can be characterized, reported, or integrated into interventions more specifically and accurately [22]. Including the clusters that the identified BCTs belong to at this stage will support a more efficient process of clustering BCTs for further examination against the ABACUS scale [15]. The BCTTv1 was developed to provide a standardized terminology when classifying active intervention components as BCTs. In total, the BCTTv1 identifies 93 BCTs that are distinct, nonredundant and precise, and these are further organized into 16 clusters, with the BCTs in each cluster proposed to influence behavior through a similar causal pathway (Figure 2) [22]. Figure 2 provides an example of how the BCT taxonomy [22] will be used to code BCTs within a given app and organize them into their corresponding clusters.

Reviewers will also pilot BCTTv1 coding before use on the included mobile apps to ensure that an acceptable level of interrater agreement is achieved prior to this review (>0.80) [35]. This process will be completed on two apps that are not included in this review. If required, a “codebook” will be developed to clarify coding decision rules in relation to specific features of the apps.

### Mobile App Quality

The MARS will be used to appraise the quality of each app included in the review (Multimedia Appendix 4). The MARS measures four objective domains: engagement, functionality, aesthetics, and information. It also measures a subjective rating of quality. It contains a further subjective rating for app-specific perceived impact on targeted health behaviors (although this is not included in the total score). The total score and the four objective domains have high internal consistency, suggesting that the total score provides a reliable mHealth quality rating. This also suggests that the individual domains provide reliable measures of the quality of their targeted app components (eg, engagement) [27].

Two reviewers (MM and PV) will independently apply the 23-item MARS to the included mobile apps [27]. The MARS is a reliable, simple, highly cited, and widely applicable tool designed to assess the quality of mHealth interventions [27]. It was developed and piloted in a systematic manner by an expert multidisciplinary team that included both health professionals and mHealth developers. Both reviewers will complete MARS outcome training, in line with the recommendations of Stoyanov and colleagues [27]. Reviewers will also pilot the MARS before use on the included mobile apps to ensure that an acceptable level of interrater agreement is achieved prior to this review (>0.80) [35]. This process will be completed on 2 apps.

Disagreements will be resolved by discussion, and where consensus is not possible, a third reviewer will decide (PM). Interrater reliability will be calculated for the MARS total score and for all four individual objective domains between the reviewers using an identical method to the BCT coding reliability analysis.

### Behavior Change Potential

The ABACUS will be used to evaluate the behavior change potential of the included apps (Multimedia Appendix 5) [15]. The ABACUS assesses four BCT clusters: knowledge and information, goals and planning, feedback and monitoring, and actions [15]. As this scale provides a validated framework for the identification and quantification of the behavior change potential of mobile apps, it provides an excellent measure and fit to suit our research objectives [15]. Based on the BCTs identified in apps in earlier steps of this review, two reviewers (MM and PV) will independently score the behavior change potential. BCTs identified in apps that are not covered by the ABACUS will also be recorded to capture those not already covered as part of the ABACUS outcome.

In line with the protocols for application of the BCT coding and MARS instrument scoring, the reviewers will pilot the ABACUS before use on the included mobile apps to ensure that an acceptable level of interrater agreement is achieved prior to this review (>0.80) [35]. This process will be completed on two apps.

Disagreements will be resolved by discussion, and where consensus is not possible, a third reviewer will decide (JF).

### Data Synthesis

As outlined in Textbox 3, app characteristics will be organized as proportion (percentage) for categorical data. This will include BCTs (and the behaviors they are targeting) and their corresponding clusters identified in the included apps; involvement of a health care professional in development (yes/no; role); the cost category (free, app purchase or in-app purchases); the platform (Apple App Store, Google Play, or both); the top apps for MARS total score as well as for the individual objective MARS domain scores; the top-scoring apps according to the ABACUS; and app focus (specific condition/painful region, or more general; specialty).

A matrix will be used to visualize the BCTs used to target different behaviors. Continuous data will be presented both as individual scores for each included app and also as mean (standard deviation), such as cost amount and consumer review ratings.

## Results

The app store search and screening are expected to be completed in 2021. Data extraction and quality appraisal are expected to commence by November 2021. The study results are expected to be published in a subsequent paper in 2022.

## Discussion

### Expected Findings and Interpretations

Despite the potential of apps to support the delivery of physiotherapy care, there is little information about the range, content, and quality of mobile apps available for consumers in app stores. Understanding about (1) what apps are available, (2) the BCTs they use, (3) their quality, and (4) their potential to change behavior is still in its infancy. Our systematic review of apps in app stores will lay the groundwork to address these gaps, helping to identify limitations of currently available apps and providing guidance for researchers and app developers about how to improve or expand mobile apps intended for use by patients to better support physiotherapy care.

This review will also allow for comparison of the behaviors targeted by apps with the behaviors that physiotherapists routinely aim to target when delivering care [1]. Ultimately, the findings of this review will determine the likely utility of such apps for supporting physiotherapy care in patient users. At present, it should be noted that there is no agreed-upon validated overall MARS or ABACUS score that infers validity of these apps [15,27,36]. A recent study was conducted to validate the MARS scale [37]. This study combined mobile app quality data from several international MARS reviews, suggesting that overall MARS quality was moderate across a range of mobile health apps (mean score 3.74/5). The study further presents variable data for average quality scores across all domains of the MARS. This finding is echoed by the creators of the ABACUS [15]. The ABACUS does not presently propose to suggest a correlation between score and outcomes [15]. In a validation study of the ABACUS, the authors present an average score of 7.8 out of 21 for mobile health apps, suggesting a low to moderate number of BCTs in these apps. They suggest that the implications of the relative scores of apps remain to be ascertained. This is required to understand and infer the relative importance of these ratings. Although no final agreed scoring cutoffs are recommended, research may benefit from comparison against these mean scores [15,37].

Future research may involve a systematic literature review to evaluate the evidence for the efficacy of the apps that we identify. This research may seek to analyze the multiple features of mobile apps and their relationship to BCTs and clinical outcomes, to guide recommendations for their use. We further envision that using the findings from our review, physiotherapy researchers may be able to consider the mean quality and behavior change potential scores derived as a benchmark for apps in the physiotherapy care arena. The knowledge gained from this review will also support further clinical research, including formal randomized evaluations that use mobile app–based interventions, or potential enhancement of existing apps. It may also inform decision-making about whether to integrate apps into patient care in clinical practice. Importantly, this review will also identify market gaps that can stimulate future research in the development and evaluation of evidence-based and BCT-informed mobile apps.

### Limitations

Although this study protocol is grounded in well-established and evidenced methods for searching, extracting, and evaluating information from within mobile apps in app stores, it is not without limitation. The proposed search strategy is designed to capture the most commonly searched and downloaded apps, while retaining feasibility by limiting the number of apps retrieved with each search to the first 100 [25]. Considering that in total, 210 searches will be performed (10 in step one and 200 in step two), as many as 21,000 apps could foreseeably be retrieved despite the limit. Furthermore, in developing the search strategy, we deliberately sought to include only apps that pertain to physiotherapy or physical therapy to further enhance the feasibility and specificity of the retrieval. Without this targeting, it is likely that the search will yield thousands of apps that are not relevant to physiotherapy care. However, we acknowledge that it is possible that our targeted selection criteria (that exclude apps that do not specifically mention physiotherapy) may miss some apps that physiotherapists recommend to patients*.* This review also follows other similar published systematic review research into health-based apps within app stores published in this journal, which identified 6579 apps [25]. It is possible that this cap limits other apps that would have been included without a search limit. However, based on this published approach [25] that included 18 apps for full analysis, we anticipate we will identify a similar number using our pre-selected terms. Additionally, to enhance coverage and to ensure that the retrieved apps are relevant to the outcomes of this study are not missed, we plan to search the websites of professional physiotherapy associations for any mobile apps that may be recommended for use by patients to support physiotherapy care. As this systematic review of apps in app stores is not a systematic review of published literature, we will not be able to draw conclusions about the efficacy of the apps for changing patient behaviors or for improving outcomes of physiotherapy care. This may be an area to target for future research.

## Appendix

Multimedia Appendix 1 List of World Physiotherapy member organizations to be searched for recommended mobile apps.

Multimedia Appendix 2 Descriptive data extraction spreadsheet.

Multimedia Appendix 3 Behavior change technique coding spreadsheet.

Multimedia Appendix 4 Mobile Application Rating Scale data extraction spreadsheet.

Multimedia Appendix 5 App Behaviour Change Scale data extraction spreadsheet.

## Abbreviations

ABACUS: App Behavior Change Scale
BCT: behavior change technique
BCTTv1: Behavior Change Technique Taxonomy version 1
MARS: Mobile Application Rating Scale
mHealth: mobile health
PRISMA: Preferred Reporting Items for Systematic Reviews and Meta-Analyses
PRISMA-P: Preferred Reporting Items for Systematic Review and Meta-Analysis Protocols
