# Supplementary material for: Patient-Facing Mobile Apps to Support Physiotherapy Care: Protocol for a Systematic Review of Apps Within App Stores
Source: JMIR Res Protoc. 2021 Dec 9;10(12):e29047. doi: 10.2196/29047 (PMC8704116; doi:10.2196/29047)
Supplement: Multimedia Appendix 1 [file resprot_v10i12e29047_app1.docx]

| Organisation | Website |
| --- | --- |
| Japanese Physical Therapy Association | http://www.japanpt.or.jp/english/international/for-foreigner/english/ |
| French National Council of Physiotherapists | <https://www.ordremk.fr>  *Excluded as website not available in English* |
| Polish Chamber of Physiotherapists | <https://kif.info.pl/polish-chamber-physiotherapists/>  *Excluded as website not available in English* |
| American Physical Therapy Association | https://www.apta.org |
| Chartered Society of Physiotherapy | https://www.csp.org.uk |
| Indian Association of Physiotherapists | https://www.physiotherapyindia.org |
| German Association for Physiotherapy | <https://www.physio-deutschland.de/mitglied-werden.html>  *Excluded as website not available in English* |
| Australian Physiotherapy Association | http://www.australian.physio |
| Canadian Physiotherapy Association | https://physiotherapy.ca |
| Swedish Association of Physiotherapists | <https://www.fysioterapeuterna.se/In-English/>  *Excluded as website not available in English* |
