# Supplementary material for: Patient-Facing Mobile Apps to Support Physiotherapy Care: Protocol for a Systematic Review of Apps Within App Stores
Source: JMIR Res Protoc. 2021 Dec 9;10(12):e29047. doi: 10.2196/29047 (PMC8704116; doi:10.2196/29047)
Supplement: Multimedia Appendix 5 [file resprot_v10i12e29047_app5.pdf]

ABACUS data

		1. Knowledge and information					*2. Goals and planning*			*3. Feedback and monitoring*							*4. Actions*					
App identification number	App name	1.1	1.2	1.3	1.4	1.5	2.1	2.2	2.3	3.1	3.2	3.3	3.4	3.5	3.6	3.7	4.1	4.2	4.3	4.4	4.5	4.6
1	App 1																					
2	App 2																					
