# Supplementary material for: Patient-Facing Mobile Apps to Support Physiotherapy Care: Protocol for a Systematic Review of Apps Within App Stores
Source: JMIR Res Protoc. 2021 Dec 9;10(12):e29047. doi: 10.2196/29047 (PMC8704116; doi:10.2196/29047)
Supplement: Multimedia Appendix 3 [file resprot_v10i12e29047_app3.pdf]

BCT data

BCT Reference	BCT Name	App 1	*justification*
1.1	Goal setting (behavior)		
1.2	Problem solving		
1.3	Goal setting (outcome)		
1.4	Action planning		
1.5	Review behavior goal(s)		
1.6	Discrepancy between current behavior and goal		
1.7	Review outcome goal(s)		
1.8	Behavioral contract		
1.9	Commitment		
2.1	Monitoring of behavior by others without feedback		
2.2	Feedback on behavior		
2.3	Self-monitoring of behavior		
2.4	Self-monitoring of outcome(s) of behavior		
2.5	Monitoring outcome(s) of behavior by others without feedback		
2.6	Biofeedback		
2.7	Feedback on outcome(s) of behavior		
3.1	Social support (unspecified)		
3.2	Social support (practical)		
3.3	Social support (emotional)		
4.1	Instruction on how to perform a behavior		
4.2	Information about antecedents		
4.3	Re-attribution		
4.4	Behavioral experiments		
5.1	Information about health consequences		
5.2	Salience of consequences		
5.3	Information about social and environmental consequences		
5.4	Monitoring of emotional consequences		
5.5	Anticipated regret		
5.6	Information about emotional consequences		
6.1	Demonstration of the behavior		
6.2	Social comparison		
6.3	Information about others' approval		
7.1	Prompts/cues		
7.2	Cue signalling reward		
7.3	Reduce prompts/cues		
7.4	Remove access to the reward		
7.5	Remove aversive stimulus		
7.6	Satiation		
7.7	Exposure		
7.8	Associative learning		
8.1	Behavioral practice/ rehearsal		
8.2	Behavior substitution		
8.3	Habit formation		
8.4	Habit reversal		
8.5	Overcorrection		
8.6	Generalisation of a target behavior		
8.7	Graded tasks		

BCT Reference	BCT Name	App 1	*justification*
9.1	Credible source		
9.2	Pros and cons		
9.3	Comparative imagining of future outcomes		
10.1	Material incentive (behavior)		
10.2	Material reward (behavior)		
10.3	Non-specific reward		
10.4	Social reward		
10.5	Social incentive		
10.6	Non-specific incentive		
10.7	Self-incentive		
10.8	Incentive (outcome)		
10.9	Self-reward		
10.1	Reward (outcome)		
10.11	Future punishment		
11.1	Pharmacological support		
11.2	Reduce negative emotions		
11.3	Conserving mental resources		
11.4	Paradoxical instructions		
12.1	Restructuring the physical environment		
12.2	Restructuring the social environment		
12.3	Avoidance/reducing exposure to cues for the behavior		
12.4	Distraction		
12.5	Adding objects to the environment		
12.6	Body changes		
13.1	Identification of self as role model		
13.2	Framing/reframing		
13.3	Incompatible beliefs		
13.4	Valued self-identity		
13.5	Identity associated with changed behavior		
14.1	Behavior cost		
14.2	Punishment		
14.3	Remove reward		
14.4	Reward approximation		
14.5	Rewarding completion		
14.6	Situation-specific reward		
14.7	Reward incompatible behavior		
14.8	Reward alternative behavior		
14.9	Reduce reward frequency		
14.1	Remove punishment		
15.1	Verbal persuasion about capability		
15.2	Mental rehearsal of successful performance		
15.3	Focus on past success		
15.4	Self-talk		
16.1	Imaginary punishment		
16.2	Imaginary reward		
16.3	Vicarious consequences		
